# Climate-based forecasting from national *Culex* mosquito surveillance to support West Nile and Usutu virus preparedness in England and Wales

**DOI:** 10.1186/s12889-026-28033-5

**Published:** 2026-06-23

**Authors:** Joanna Nevison de Klerk, Amirah Haziqah-Rashid, Emma Widlake, Roksana Wilson, Jack Pilgrim, Alexander G. C. Vaux, Jolanta Tanianis-Hughes, Agata Delnicka, Amy S. Jealous, Anthony J. Abbott, Catie Haines, Colin J. Johnston, Finn Miller, Ken Sherlock, Fatma Bursali, Sara Gandy, Sarah M. Biddlecombe, Jolyon M. Medlock, Marcus S. C. Blagrove, Matthew Baylis, Luigi Sedda

**Affiliations:** 1 Lancaster Ecology and Epidemiology Group, Lancaster Medical School, Lancaster University, Lancaster, LA1 4AT UK; 2 Institute of Infection, Veterinary and Ecological Sciences, Faculty of Health and Life Sciences, University of Liverpool, Liverpool, L69 3BX UK; 3 Medical Entomology & Zoonoses Ecology, Centre for Climate & Health Security, Health Security Agency, Porton Down, Salisbury, Wiltshire SP4 0JG United Kingdom; 4 Faculty of Science, Department of Biology, Aydin Adnan Menderes University, Aydin, 09010 Turkey

**Keywords:** Culex, West Nile virus, Spatial mapping, Usutu virus, Mosquito abundance, GLMM

## Abstract

*Culex* mosquitoes are widespread in temperate regions and play a key role in transmitting veterinary and human vector-borne diseases. In the United Kingdom, *Culex pipiens* s.l. is highly prevalent and a competent vector of West Nile and Usutu viruses. Coupled with the northward expansion of West Nile virus in Europe, this raises concerns about emergence in the UK. For public and animal health preparedness, and effective vector control planning, it is essential to better understand the distribution of *Culex* mosquitoes in this area.

This study developed species distribution maps for *Culex pipiens pipiens*, *Culex pipiens molestus*, and *Culex torrentium* abundance using data from the first nationwide stratified active mosquito surveillance programme in England and Wales in 2023, supplemented with adaptive surveillance in 2024. *Culex p. pipiens* models predicted higher abundances than the other two taxa. Regions of high abundance occurred across most of England, apart from the northwest, with the highest in eastern regions and estuarine areas. In contrast, higher elevation areas, including most of Wales, the North Pennines, and Yorkshire Dales, showed markedly lower abundances. Environmental drivers differed between forms. *Culex p. pipiens* abundance was strongly associated with precipitation-related covariates, whereas *Culex p. molestus* was mostly influenced by temperature covariates. These findings highlight the importance of modelling the two forms separately in risk analyses and distribution studies.

The resulting models provide timely ecological insights to support surveillance prioritisation and provide a foundation for future work aimed at guiding public health planning and targeted vector management.

**Supplementary Information:**

The online version contains supplementary material available at https://doi.org/10.1186/s12889-026-28033-5.

## Introduction

Mosquitoes of the *Culex* genus pose major veterinary and human public health threats as vectors of high impact diseases. They are widespread in temperate regions [1] and are responsible for the transmission of viruses such as West Nile virus (WNV) [2], Usutu virus [3], Japanese encephalitis virus [4], St. Louis encephalitis virus [5] and Eastern and Western equine encephalitis viruses [6, 7]. Critically, the generalist feeding behaviour of some *Culex* species on birds and mammals, including humans [8], enables them to act as bridge vectors, driving the spillover of zoonotic pathogens into human populations.

Mosquito-borne diseases have the potential to pose a meaningful challenge for the United Kingdom, that are likely to be similar to those already experienced in parts of Europe. If an outbreak of WNV, a zoonotic mosquito-borne disease outbreak, was to occur in urban areas, large numbers of people could potentially be exposed. The elderly (over 65), which comprise almost one fifth of the population [9], are particularly vulnerable, with mortality and severe disease outcomes disproportionately higher compared to younger individuals [10]. The introduction and/or establishment of such pathogens would not only threaten individual health but also compound existing population-level health burdens such as chronic diseases like cancer, cardiovascular conditions [11], and the long-term consequences of COVID-19 [12]. The potential combination of these stressors highlights an urgent need to anticipate and mitigate high impact mosquito-borne disease risk in the UK.

Across England and Wales, *Culex pipiens* s.l. mosquitoes are highly prevalent and are found in urban, peri-urban and rural areas, while *Culex torrentium* mosquitoes have a preference for rural areas [13]. They are both competent vectors for WNV as in the rest of Europe [14]. WNV is a flavivirus which is transmitted in a sylvatic cycle between mosquitoes and predominantly passerine birds and can spill over into human and equine populations, which are dead-end hosts. Although the UK has not faced endemic human mosquito-borne diseases in the past century prior to the emergence of Usutu virus, the risk of arbovirus emergence is real, as climate change such as rising temperatures and altered precipitation patterns, expand the distribution, abundance, and seasonal activity of vectors [15, 16], and create the potential for significant future outbreaks aided by the intensification of human activity and changes in land use [17–19]. In recent years, the expansion of WNV northwards in continental Europe has been observed from the countries in the Mediterranean Basin [20], which peaked during 2018–2022, mostly in Italy and Greece, to Germany and The Netherlands [21]. In 2020, for the first time, nine human WNV cases were observed in Germany where six patients showed neurological disease and one patient died, whilst concurrently, six WNV cases were reported in The Netherlands with two exhibiting severe neurological disease [21]. This raises concerns about imminent emergence in the United Kingdom, particularly since the virus was detected for the first time in pools of mosquitoes from 2023 surveillance activities [22]. Additionally, Usutu virus continues to spread across Europe [23], and more recently in the UK, where it has been detected in blackbirds and house sparrows in Greater London in 2020 and every year since [24], and in a wide range of mosquito species [25] highlighting the ability for these viruses to be introduced, and potentially circulate where they have historically been absent.

Understanding the current distribution of *Culex* mosquitoes in England and Wales, and how it might change in the future, is crucial for supporting surveillance prioritisation, and providing a foundation for further work which may contribute to public and animal health preparedness and vector control planning. Previous mosquito-borne disease work in the United Kingdom used species distribution models (SDMs) to produce spatially explicit output maps estimating the likelihood of combined species of mosquitoes competent for transmitting Rift Valley fever (*Anopheles claviger*, *Anopheles messeae*, *Anopheles atroparvus*, *Culiseta annulata*, and *Aedes detritus*) [26]. Additionally, the probability of presence of *Aedes albopictus* and *Aedes japonicus* have been modelled for the whole of Europe [27] and the UK [28], as well as the seasonal abundance of *Aedes albopictus* in Europe [29]. However, there remains a lack of detailed, region-specific modelling for *Culex* mosquitoes in the United Kingdom, both for present day and future scenarios based on national systematic surveillance data. A recent publication employed presence models using historical data up until 2022 from the National Biodiversity Network (NBN) Atlas and VectorNet data [30] in the UK, however, models for both presence and abundance based on active mosquito surveillance are yet to be developed. Addressing this gap is vital, given the relevance of these vectors for transmission of important arboviruses, including maintaining circulation and acting as bridge vectors for spillover into human populations. In this context, our work represents a significant advance. For the first time, we build models on nationally comprehensive data stratified by land cover, an approach not previously available, and extend these models to project future scenarios. Such models are essential for guiding targeted vector surveillance, improving risk assessment frameworks, and supporting proactive mitigation strategies in response to a changing disease landscape.

In this study, we used data collected from the first nationwide stratified adaptive active mosquito surveillance programme in England and Wales in 2023 and 2024, to develop species distribution maps for the abundance of *Culex pipiens pipiens*,* Culex pipiens molestus*, and *Culex torrentium* [13], where abundance is defined as the predicted number of mosquitoes caught by our sampling methodology in a 72 h period. We assumed that the surveillance data reflects species distributions, and that species-environment relationships remain consistent over time, including under future climate scenarios. The aim was to produce *Culex p. pipiens*,* Culex p. molestus*, and *Culex torrentium* distribution models, both for present day and future scenarios, to better understand their environmental drivers and identify areas where higher mosquito abundances can or are occurring.

## Methodology

### Mosquito surveillance

During the months of July to early August in 2023 and 2024, mosquito surveillance was undertaken across England and Wales simultaneously by two teams from the University of Liverpool and the UK Health Security Agency. Two-hundred sampling points were selected for 2023 using a “lattice plus close pairs design” to ensure sufficient spatial coverage, suitable model inference, and adequate representation of the spatial processes under study. Analyses confirmed that data collected by the two teams were consistent, with no detectable team-related bias across the surveyed regions. Traps were set for run for 72 h in each location which was not resurveyed in the same year. This design and trapping methods of this initial 2023 surveillance are described fully in our previous work [13].

In 2024, an adaptive sampling method based on spatial generalised linear modelling and theory of extremes [31] was adopted to reduce the uncertainty of the mosquito estimates and increase *Culex* catches. An additional 98 sites were identified, which were ranked on priority based on the effect the sample location would have on the model uncertainty, as well as the ability to maximise mosquito collections. The top 59 sites were then chosen, 44 of which were repeat sites from 2023, and 15 were new sites, with the remainder available in case any sites were inaccessible.

Mosquitoes of the *Cx. pipiens* complex were identified morphologically, while molecular techniques were used to identify *Cx. pipiens* biotype *pipiens*, *Cx. pipiens* biotype *molestus*, *Cx. pipiens/molestus* hybrids and *Cx. torrentium* [13], however hybrids were not used in the development of the abundance models presented in this study due to small sample sizes. Nevertheless, hybrid locations have been plotted in the Supplementary Material.

### Environmental covariates

Environmental data were downloaded from Copernicus Climate Data Store, WorldClim 2.1 and the United Kingdom Centre for Ecology and Hydrology (UKCEH) (source references are available in the Supplementary Material). Once compiled, each numerical environmental raster was cropped to England and Wales, and scaled between 0 and 1, using min-max scaling of the combined 2023 and 2024 values. These same minimum and maximum values were then applied to scale the future covariate raster values. The list of all environmental covariates, their abbreviations and descriptions and the pre-processing steps are presented in the Supplementary Material.

### Model selection

All analysis was undertaken using R (version 4.4.1; R Core Team, 2025) and RStudio (version 2025.05.1 + 513 “Mariposa Orchid” Release). After removal of colinear covariates using a Pearson’s correlation matrix (full methodology described in the Supplementary Material), the remaining list of candidates was then used to analyse all combinations of two or more covariates for each species model. A k-fold cross validation approach was then used to iterate through each covariate combination, using three randomisations of subsets of testing and training data. Data were subsetted into testing (20%) and training (80%) data, where the testing dataset contained pairs of points of which distances were representative of the spatial autocorrelation described by the exponential variogram of the outcome [32].

The predictive performance of each model was measured using the normalised residual mean square error (nRMSE) metric, normalised by dividing by the mean.

Models for confirmed *Cx. p. pipiens*, *Cx. p. molestus* or *Cx. torrentium* abundance collected during this study at each location were fitted by using generalized linear mixed models (GLMM) with a negative binomial distribution where variance increases quadratically with the mean (negative binomial type II) and a log link function, implemented in the *glmmTMB* package [33] expressed by,

$$\log\left(\mu_i\right)=\beta_0+\sum_{j=1}^k\beta_j\;\ast\;x_{i,j}\;+\;\omega_{u\left(i\right)}$$

where $\:{\mu\:}_{i}$ is the expected mosquito abundance at site * i*, $\:{\beta\:}_{0}$ is the intercept of the model, $\:{\beta\:}_{j}$ is the coefficient associated with covariate * j*, $\:{x}_{i,j}$ is the value of the covariate * j* at site * i*, and $\:{\omega\:}_{u\left(i\right)}\:\sim\:N(0,{\sigma\:}_{\omega\:}^{2})$ represents the random effect associated with spatial grouping. Since the response variable was assumed to follow a negative binomial distribution (type II), which inherently accounts for overdispersion, no separate independent error term was included.

The mean of the nRMSE values across the three iterations were then calculated for each combination of two or more covariates, and then models were ordered from lowest to highest nRMSE. Each model was analysed in turn for zero-inflation, under/overdispersion and deviations from uniformity of simulated residuals and was rejected if found to be significant for any of these statistics. Additionally, the model required at least one significant covariate to be accepted. These were determined to be the acceptance criteria and continued until 100 models had been accepted to build a mean ensemble. An ensemble approach was used to reduce model selection bias and overfitting associated with reliance on a single optimal model. This approach improves robustness by averaging across multiple plausible model structures and formulae. Despite this improvement in robustness to individual model choice, shared assumptions across candidate models mean that results should be interpreted primarily in a predictive, rather than strictly inferential, context. To test for spatial autocorrelation, Moran’s I was analysed, and if significant, a spatial cluster ID group was added as a random effect. The spatial group was developed through hierarchical clustering of data points, using the R function *hclust*, under 70 km, 80 km, 90 km and 100 km distance thresholds (under 70 km was not used as otherwise there would be less than two data points in each cluster). In all cases, the 70 km spatial group performed the best when comparing the AIC and BIC metrics of the models, which consistently worsened the larger the distance threshold.

The final chosen model ensembles were then used to project raw abundance maps, for present day (mean of 2023 and 2024 real-world data), as well as 2030, 2050, 2070 and 2090 under RCP 4.5 and RCP 8.5 predicted climate scenarios. To improve the map visualisation and inference, abundance was log_10_-transformed (where, 0 = 1, 1 = 10, 2 = 100, etc.), after adding 1 to the abundance to avoid negative infinity values for zero abundances, or negative values for abundance predictions < 1 when taking the logarithm. Areas where values were above the 99th percentile were capped to prevent presentation of spurious results. Maps with capped areas masked out are presented in the Supplementary Material.

The same analyses were then repeated using a binomial model to explore the distribution of presence and absence of *Culex* mosquitoes and are presented in the Supplementary Material.

The primary objective of this modelling framework was to optimise predictive performance and spatial generalisation rather than to identify a single statistically optimal or causally interpretable model. As such, inference regarding individual covariate effects should be interpreted cautiously, and the framework is best understood as a predictive, rather than confirmatory, modelling approach.

## Results

Models were developed from extensive mosquito catches in 2023 and 2024 and built upon the following data in Table 1.

**Table 1:** Summary statistics for mosquito collections by species and year

Species	Year	Total caught	Total overall	Mean catch	Mean overall	Standard deviation	Standard deviation overall
*Cx. p. pipiens*	2023	1478	2873	7.4	11.1	22.1	57.7
	2024	1395		24.1		114.5	
*Cx. p. molestus*	2023	5	38	0.03	0.1	0.2	1.1
	2024	33		0.6		0.2	
*Cx. torrentium*	2023	38	52	0.2	0.2	0.7	0.7
	2024	14		0.2		0.8	

All model ensembles were developed from the top 100 performing models which met the acceptance criteria as outlined in the methodology, out of an exhaustive search of 8,178 models for *Cx. p. pipiens* and *Cx. p. molestus*, and 2,036 models for *Cx. torrentium* abundance. These final chosen 100 models were then evaluated using a five-fold cross validation approach, with random effects now added where required, which showed the *Culex p. pipiens* models included in the ensemble had a mean nRMSE of 3.51 ± 1.04, the *Cx. p. molestus* models had a mean nRMSE of 9.47 ± 6.12, and the *Cx. torrentium* models had a mean nRMSE of 3.77 ± 1.14.

**Fig. 1:**
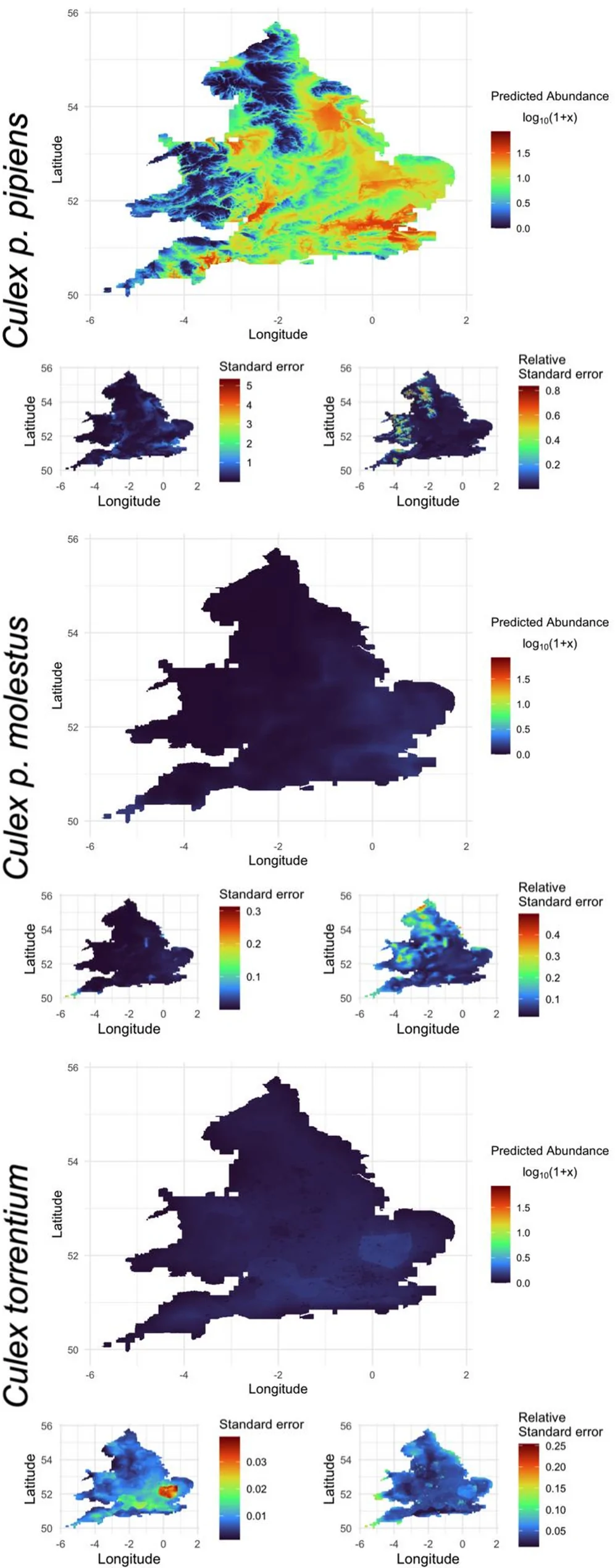
Projected *Cx. p. pipiens*, *Cx. p. molestus* and *Cx. torrentium* log_10_-transformed abundances for present day (mean 2023 and 2024 ERA-5 climate data), with below each map, standard error of prediction of the model (left) and coefficient of variation displaying relative uncertainty of the model (standard error/mean) (right). Scales for log_10_-tranformed abundance maps have been made consistent to directly compare the differences. Standard error and relative standard error maps are presented having been calculated from non-transformed abundance values. Spatial artefacts observed from cluster boundaries in the *Culex torrentium* maps are described in the Supplementary Material.

Figure 1 illustrates the spatial distribution and abundance levels of all three species for comparative purposes, as well as the uncertainty of the models. *Culex p. pipiens* had the highest abundance of all species (0 to 83 individual mosquitoes, or 0 to 1.9 when $\:{log}_{10}(x+1)$ transformed), with generally high numbers across most of England, apart from the northwest (Fig. 1). The highest abundances were around estuaries, such as the Thames, Exe, Severn and Humber estuaries, as well as in the east, such as Yorkshire, Cambridgeshire and Greater London (maps with geolocations provided in the Supplementary Material). Areas of higher elevation were estimated to have lower abundances, such as Wales, the North Pennines and Yorkshire Dales. *Culex p. molestus* and *Cx. torrentium* demonstrated comparatively much lower abundances than *Culex p. pipiens* overall. *Culex p. molestus* was predicted to be slightly higher in relative abundance in the southeast compared to other areas, and follow a similar spatial pattern of low abundance to *Cx. p. pipiens*, however these results should be considered exploratory due to the sparsity and low catches of *Culex p. molestus*. *Culex torrentium* was predicted to have a higher abundance in the south of England, but with lower abundance in urban areas.

Present day model predictions captured the spatial variation of field observations well, although the ranges of abundance were less reliable (as illustrated by the scatterplots in the Supplementary Material). Both abundance and probability of presence maps with field data overlaid are available in the Supplementary Material.

For all three species, predicted abundance decreased with increasing elevation and increasing latitude (Table 2). Mapped predictions for present day in both *Cx. p. pipiens* and *Cx. p. molestus* models demonstrated higher model relative uncertainty in areas of lower predicted abundance in the north of England and Wales, and *Cx. torrentium* had a general low relative uncertainty apart from in areas with high densities of buildings and some coastal areas in the southwest and northwest (Fig. 1).

**Table 2:** Comparison between mean predicted raw abundance (and log_10_(x + 1) transformed abundance in brackets) across all pixels in that category and elevation/latitude. In all species, abundance decreased with increasing elevation or latitude

Elevation	Mean Predicted Abundance		
	*Culex p. pipiens*	*Culex p. molestus*	*Culex torrentium*
< 0 m	16.91 (1.25)	0.28 (0.11)	0.29 (0.11)
0–100 m	12.31 (1.12)	0.16 (0.06)	0.22 (0.09)
100–200 m	4.12 (0.71)	0.07 (0.03)	0.23 (0.09)
200–300 m	0.99 (0.30)	0.02 (0.01)	0.18 (0.07)
300–400 m	0.31 (0.12)	0.01 (< 0.01)	0.17 (0.07)
400–500 m	0.13 (0.05)	< 0.01 (< 0.01)	0.16 (0.06)
500–600 m	0.07 (0.03)	< 0.01 (< 0.01)	0.16 (0.06)
600–700 m	0.05 (0.02)	< 0.01 (< 0.01)	0.14 (0.06)
700–800 m	0.04 (0.02)	< 0.01 (< 0.01)	0.13 (0.05)
800–900 m	0.04 (0.02)	< 0.01 (< 0.01)	0.12 (0.05)
900–1000 m	0.05 (0.02)	< 0.01 (< 0.01)	0.12 (0.05)

Latitude			
50–51°N	7.18 (0.91)	0.15 (0.06)	0.20 (0.08)
51–52°N	10.50 (1.06)	0.15 (0.06)	0.28 (0.11)
52–53°N	7.67 (0.94)	0.13 (0.05)	0.24 (0.09)
53–54°N	8.85 (0.99)	0.08 (0.03)	0.16 (0.06)
54–55°N	3.52 (0.66)	0.02 (0.01)	0.13 (0.05)
55–56°N	2.19 (0.50)	0.02 (0.01)	0.12 (0.05)

From the models included in the ensemble for each species, the frequency of significant covariates was calculated (Table 3). *Culex p. pipiens* abundance was predominantly associated with precipitation covariates, and *Cx. p. molestus* abundance was predominantly associated with temperature covariates, highlighting the need to model abundance of these two *Cx. pipiens* forms separately. These results are consistent with other European studies identifying temperature and precipitation covariates being dominant significant predictors of *Culex pipiens* [34–37]. Precipitation in the month prior to collection was strongly positively associated with *Cx. p. pipiens* abundance, possibly indicating the presence of rain-filled containers and standing water suitable for breeding. The differences between *Culex p. pipiens* and *Culex p. molestus* suggest their ecology appears sufficiently separated not to be concerned about potential seasonal competition. *Culex torrentium* abundance was driven by a range of covariates, including temperature, precipitation, wind and building density.

**Table 3:** Significant environmental covariates identified by the abundance models used in the ensemble predictions and their association with abundance. A weak association (- or +) is where 50–70% of the models experienced a positive/negative association, a moderate association (-- or ++) is 70–90% and a strong association (--- or +++) is > 90%. * Percentage calculated as the percentage of times the covariate has been found significant in the ensemble formulae list

Model	Covariates	Percentage of models significant*	Association with abundance
*Culex p. pipiens*	Elevation (DEM) Mean precipitation warmest quarter (BIO18) Precipitation rate one month prior (PREC_1m) Wind speed one month prior (WND_1m) Precipitation seasonality (BIO15) Precipitation coldest month (BIO14) Number of buildings within 500 m radius (BUILD_500m) Temperature seasonality (BIO04)	98% 80% 39% 37% 4% 1% 1% 1%	--- --- +++ --- --- ++ ++ +
*Culex p. molestus*	Maximum temperature (MAX_TEMP) Mean temperature coldest quarter (BIO11) Maximum temperature on month prior (MAX_TEMP_1m) Precipitation of Driest Quarter (BIO17) Mean temperature driest quarter (BIO09) Mean Diurnal Range (BIO02) Mean soil moisture driest quarter (SOIL_DRY)	46% 28% 19% 10% 8% 8% 3%	+++ +++ --- --- +++ +++ --
*Culex torrentium*	Temperature annual range (BIO07) Precipitation coldest month (BIO14) Wind speed one month prior (WND_1m) Number of buildings within 500 m radius (BUILD_500m) Mean precipitation warmest quarter (BIO18) Maximum temperature (MAX_TEMP)	52% 25% 20% 8% 3% 2%	+++ --- --- --- -- ---

**Fig. 2:**
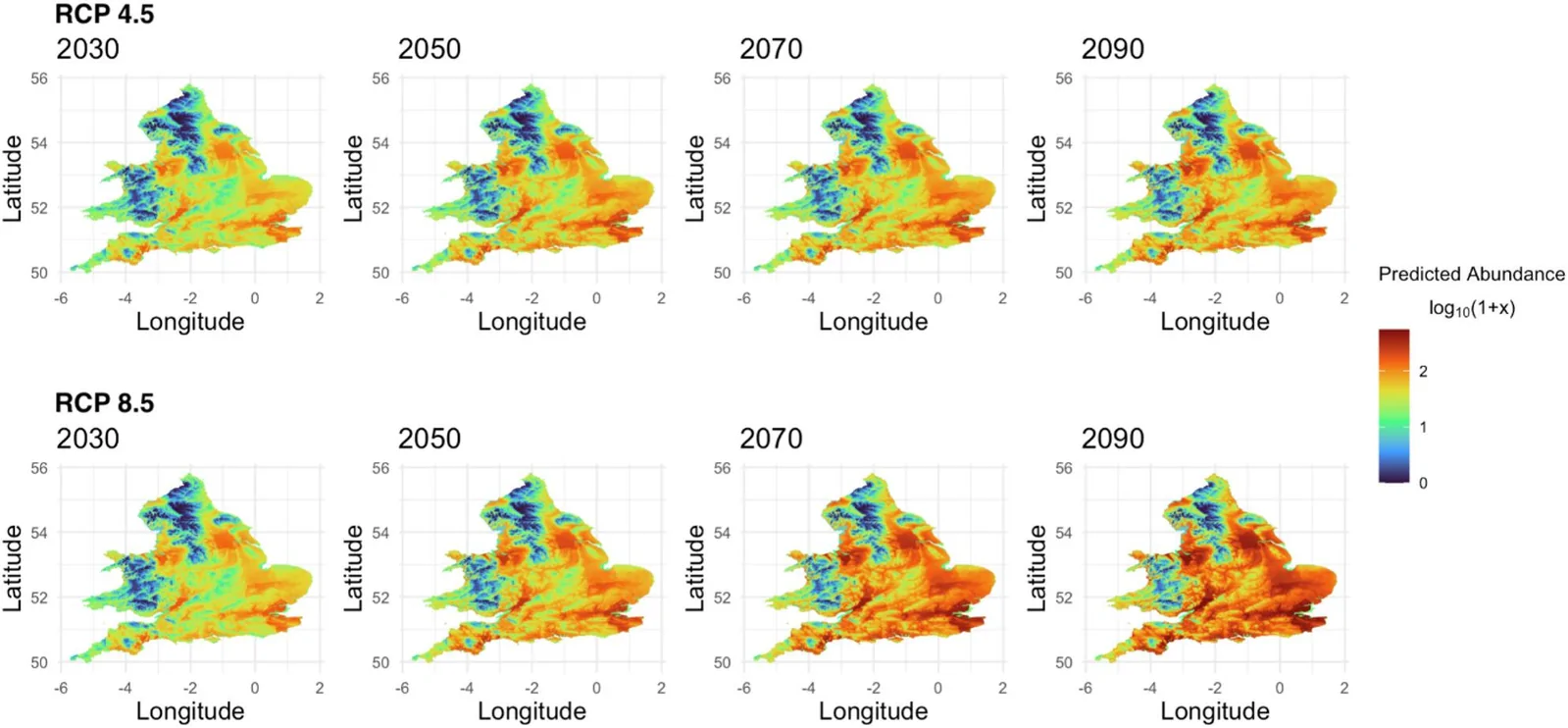
Future projections of Cx. p. pipiens log_10_-transformed abundance under the RCP 4.5 and RCP 8.5 pathways demonstrated a mild increase in abundance in areas of high abundance in the RCP 4.5 pathway and a marked increase in abundance in areas of high abundance in the RCP 8.5 pathway

**Fig. 3:**
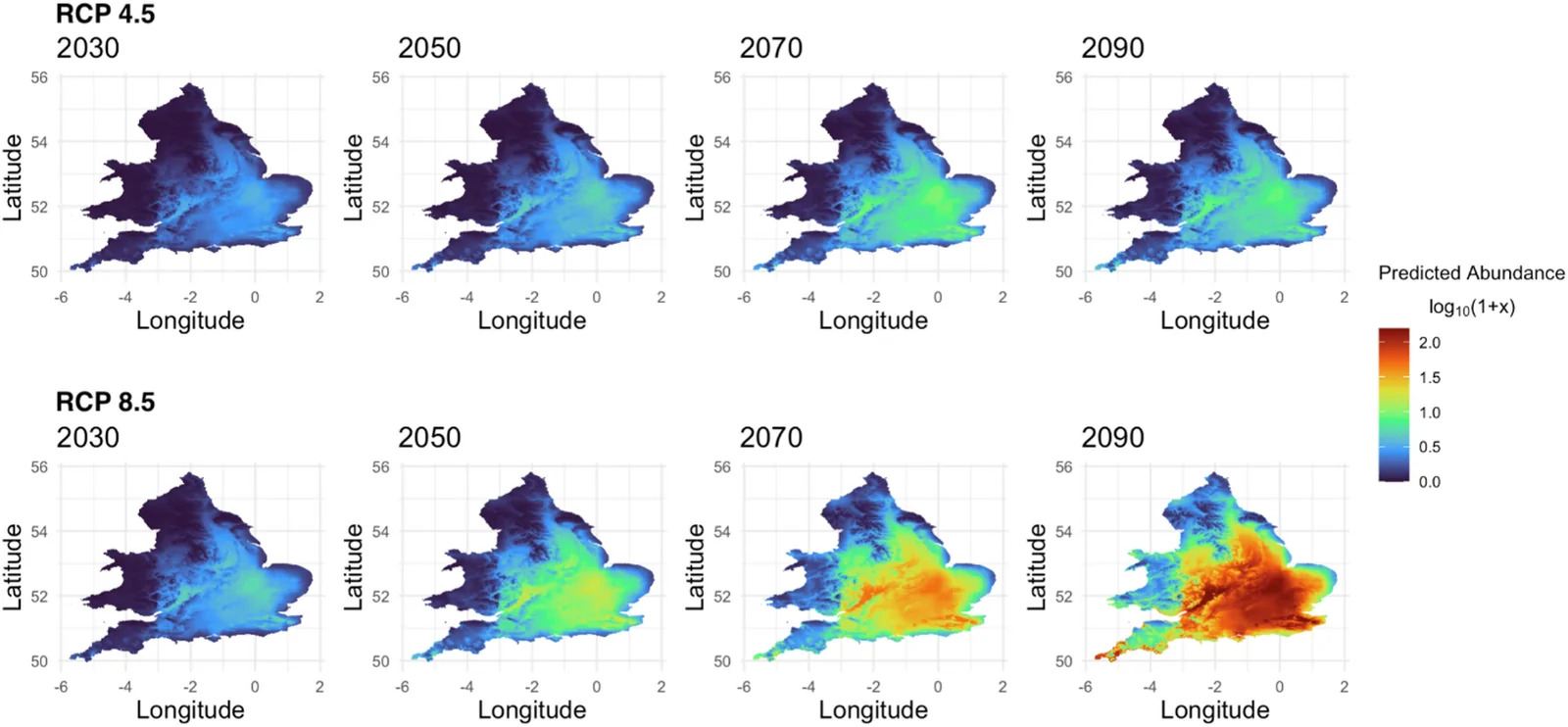
Future projections of Cx. p. molestus log_10_-transformed abundance under the RCP 4.5 and RCP 8.5 pathways demonstrated a mild increase in abundance in the southeast in the RCP 4.5 pathway and a marked increase in abundance in the southeast in the RCP 8.5 pathway

Future mapped predictions for climate scenarios RCP 4.5 and RCP 8.5 showed mild to marked changes in the *Cx. p. pipiens* and *Cx. p. molestus* populations (Figs. 2 and 3), predicting an increase in abundance throughout the century, particularly dramatically in the RCP 8.5 scenario for both forms of the species. RCP 4.5 represents a moderate emissions scenario where greenhouse gas concentrations stabilise by the end of the century, whereas RCP 8.5 reflects a high emissions scenario characterised by continued increases in greenhouse gas emissions.

**Fig. 4:**
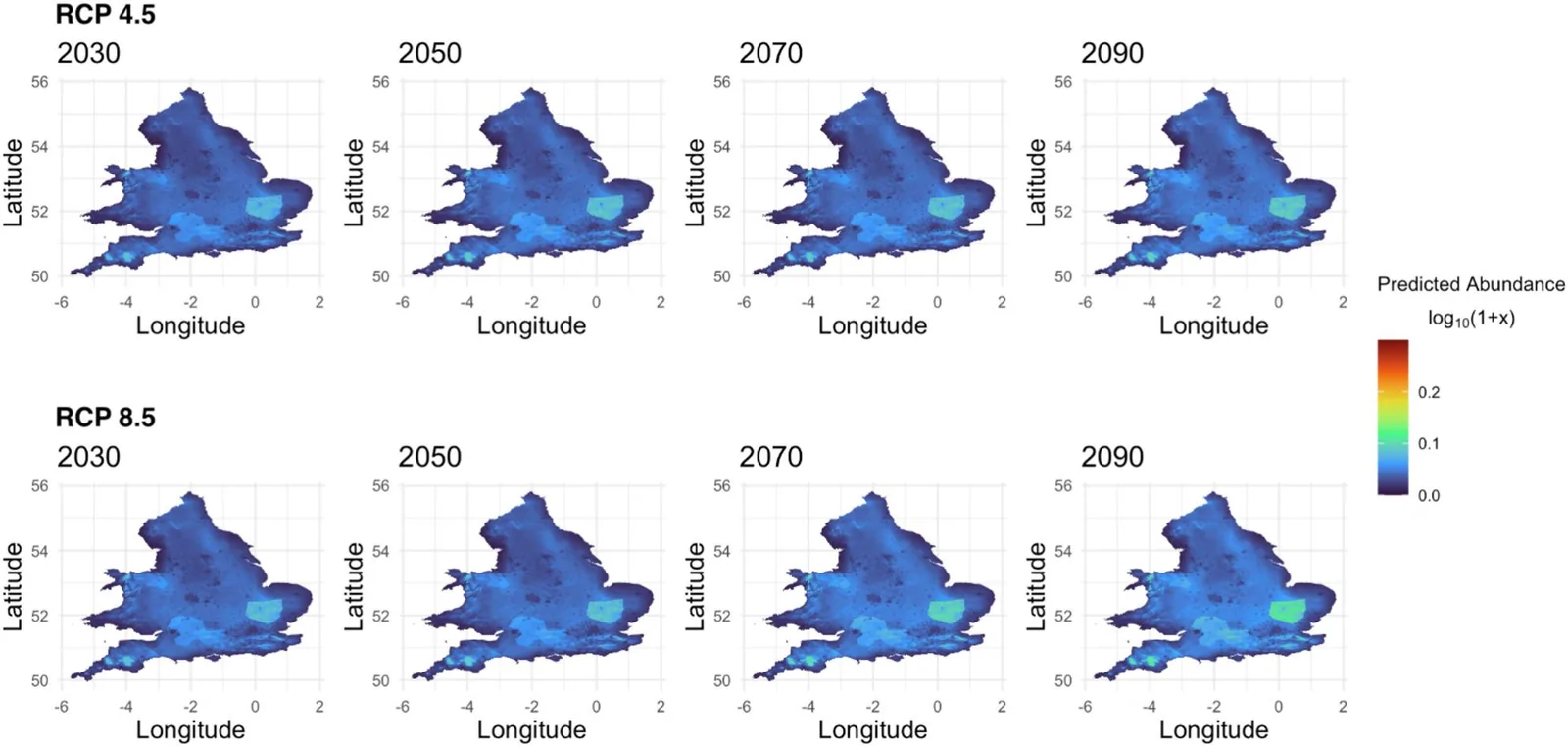
Future projections of Cx. torrentium log10-transformed abundance under the RCP 4.5 and RCP 8.5 pathways demonstrated a very slight increase in abundance in the south and Cambridgeshire in the RCP 8.5 pathway however, almost no change in the RCP 4.5 pathway

*Culex p. pipiens* further increased in abundance in areas of already higher abundance, particularly around estuaries and easterly regions, however the spatial pattern of abundance did not alter significantly. Whereas *Cx. p. molestus* increased in the southeast of England, with a widening spatial area of higher abundance as time progressed. A coastal effect was seen in the future projections of *Cx. p. molestus*, likely driven by an artefact in the future CMIP5 BIO09 covariate (maximum temperature in the driest quarter), which showed markedly lower values along the coast but was not reflected in the present-day ERA5 BIO09 data. BIO09 appeared in 8% of candidate models and consistently exhibited a positive effect (mean coefficient = 8.07 ± 1.68). This consistent signal helps explain the coastal pattern in the projections and highlights how specific covariates can influence localised outcomes. *Culex torrentium* abundance did not change significantly over the years in either future climate scenario (Fig. 4).

For completeness, “probability of presence” models for all three species are presented in the Supplementary Material.

## Discussion

Three key findings emerged from this work. First, abundance of *Cx. p. pipiens* is likely to be higher in easterly regions of England and around major estuaries, abundance of *Cx. p. molestus* is likely to be higher in Cambridgeshire, Essex, Greater London and Kent, and abundance of *Cx. torrentium* is likely to be higher in the southern areas of England, as far north as Birmingham. Second, some variation remained unexplained, particularly in the north of England and Wales for the two forms of *Cx. pipiens*, highlighting the need for further mosquito data surveillance. And third, the modelling framework successfully captured spatial variation in mosquito abundance, providing a strong foundation to accurately explore risk of transmission of mosquito-borne diseases in future work. The framework is therefore most appropriate for identifying broad spatial patterns of relative abundance and generating hypotheses, rather than making precise quantitative or causal inferences.

The findings of this research are particularly relevant at this time, as *Culex pipiens* complex and *Culex torrentium* mosquitoes are important vectors implicated in the transmission of WNV and Usutu virus [38, 39], both of which have been isolated in the United Kingdom in recent years [24, 25]. While these pathogens underscore the potential public health consequences, the primary value of this work lies in strengthening the foundation for vector surveillance. By identifying areas of high mosquito abundance, this research supports prioritisation of surveillance efforts and resource allocation, ensuring that monitoring systems are strategically positioned. This approach enhances preparedness for possible spillover to humans and equids [40], and establishes a framework for long-term investment in surveillance infrastructure. In this way, modelling abundance contributes to proactive capacity-building, enabling public health systems to detect and respond to emerging threats more effectively, particularly in contexts such as the UK’s ageing population [9, 10], where vulnerability to infectious disease impact is heightened.

Our analysis of abundance identified clear spatial patterns alongside key relationships with environmental drivers, underscoring the value of abundance modelling for revealing ecological dynamics not captured by presence–absence data alone. For example, *Cx. p. pipiens* ensemble projection indicated a high probability of presence in the majority of England, with the highest probabilities in the southeast (Supplementary Material). Withers et al. (2025) [30] produced a spatial habitat suitability map with many similarities to our *Cx. p. pipiens* probability of presence maps presented, such as a higher probability of presence around built-up areas, such as Greater London, with an overall moderate or high probability of presence across England. However, our abundance modelling revealed additional spatial dynamics, in terms of elevated abundance across the majority of England, but particularly high in estuarine regions and the east of England. This pattern is of relevance near the Severn, Thames, Exe and the Humber, a site of international importance for birdlife [41] that could facilitate arbovirus introduction or amplification. To date, published mosquito surveillance in the UK has been undertaken in the southeast [22, 42], near points of entry [43], and examined wetland and estuarine regions across England [44–48]. Our results indicate that increasing surveillance in eastern regions may be warranted, given the elevated abundance, as well as elevated probability of presence, of *Cx. pipiens* predicted in these areas. Such efforts are already complemented by UKHSA’s ongoing Nationwide Wetland Mosquito Survey [49] and Snapshot Survey which focusses on UK wetlands [50].

Furthermore, the modelling framework presented in our study also facilitated inference on how environmental and climatic variables impact mosquito abundance and presence, offering the ability to explore how distributions may change under future climate projection pathways. In our analysis, there was a projected shift in species abundance composition in the RCP 8.5 pathway (high emissions), where there were major increases in *Cx. p. pipiens* and *Cx. p. molestus* abundances, although *Cx. p. molestus* increased at a more rapid rate. *Culex p. molestus* prefers subterranean urban environments and can remain active year-round indoors [51, 52], whereas *Cx. p. pipiens*, which predominantly lives above-ground and undergoes diapause in the colder months. Additionally, host preference further amplifies risk, as *Cx. p. pipiens* typically prefers avian hosts (although will also bite mammals), whereas *Cx. p. molestus* exhibits a preference for mammals, including humans, heightening the potential for human spillover [8, 36, 53]. Moreover, the two forms can readily hybridise, resulting in mosquitoes which exhibit intermediate traits. Such hybrids have been shown to play a critical role in enhanced WNV transmission in the United States [54] and Greece [55], sustaining viral amplification in the avian reservoir while simultaneously acting as bridge vectors to human populations [53].

Geospatial vector abundance data is instrumental for vector-borne disease risk analyses, as calculating vectorial capacity (defined as the average number of potentially infectious bites that would arise from all the mosquitoes biting a single infectious host in one day [56]) inherently relies on relative vector abundance [57]. Abundance influences the number of potentially infectious contacts between vectors and hosts, which in turn impacts transmission intensity and risk, whereas presence data alone can indicate areas where transmission is possible. Aside from calculating vectorial capacity, abundance data can also be used for reproductive number estimation, demonstrated by Di Pol et al. (2022) [58] to explore the relative risk of WNV in Europe. Finally, mathematical transmission models also require knowledge of abundance to explore outbreak dynamics, demonstrated by Fesce et al. (2023) [59] to explore WNV transmission in the Lombardy region of Italy. Ultimately, our study will now allow for these types of risk analyses to be undertaken for the entirety of England and Wales, which can inform the prioritisation of surveillance resources, guide the location of vector control interventions, and support more effective public health preparedness.

One of the main limitations of this study is the assumption that the data used to build the model is a true spatiotemporal representation of the mosquito population. *Culex p. pipiens*, and to a lesser degree, *Cx. torrentium* models were more robust than those for *Cx. p. molestus*, likely reflecting the much larger number of *Cx. p. pipiens* caught. While this could be due to true differences in abundance, it is also likely that there was sampling bias introduced which was linked to habitat preferences, particularly for *Cx. p. molestus*, which is often found in subterranean or indoor environments [52]. Notably, *Cx. p. molestus* was the only species that did not show any association with buildings (Table 3). This was unexpected given its known preference for subterranean habitats, but it underscores the methodological challenges associated with trapping. Mosquitoes were sampled using a surveillance design stratified by land cover [13] then supplemented in the subsequent year using an adaptive surveillance design as earlier described. Traps were placed in suitable habitats where available, but not indoors or subterranean, and generally in sheltered areas and near standing water. However, suitable areas were not available in all land cover types. Consequently, results from these areas may affect interpretations of climatic effects, as mosquitoes may have been unlikely to occur there regardless of climate conditions. Beyond these ecological considerations, some statistical uncertainty also arises from the model selection process. Because ensemble maps were restricted to mosquito models with at least one significant covariate, the data itself influenced which models were retained. This filtering may have increased the apparent frequency of significant covariates, which in turn could elevate the chance of false positives (type I error) i.e. selecting a variable with no association with the mosquito abundance (or presence); although this risk was reduced by considering its recurrence within the ensemble instead of a single significant model. While this approach ensured that retained models captured meaningful associations, it also underscores the importance of interpreting the ensemble results with appropriate caution. Accordingly, associations between covariates and abundance identified through the ensemble, although coherent with relevant literature, should be interpreted as indicative rather than definitive since they are sensitive to field sampling bias.

In addition, the range of many future covariates, particularly under the RCP 8.5 scenario in more distant years, extends beyond the values represented in the ERA5-land covariates used to develop the models. As a result, some extrapolation was required, which should be considered when interpreting results for more extreme or long-term scenarios, as reliability may be reduced in those cases which also will require caution when interpreting.

The work presented in this study would have benefitted from repeated measurements over time, to allow the exploration of seasonal impacts on *Culex* distributions, as well as spatially, to improve inference in areas with higher uncertainty, such as the north of England. One limitation of limited trapping dates is that sampling can be heavily influenced by local environmental variability, making it challenging to distinguish true abundance from background noise. When surveillance is conducted more frequently within a season and across multiple seasons, these complex influences become better understood, and a more representative picture of abundance emerges. In this study, the addition of adaptive sampling helped reduce uncertainty of the model under cost and time constraints. Nonetheless, expanding surveillance further would enhance model certainty and strengthen inference. If further surveillance were to be carried out, trapping to target other potential UK WNV vectors could also be undertaken, such as *Aedes vexans*, in which WNV was isolated from in England [22], and other mosquito species implicated in transmission, such as *Anopheles plumbeus* Stephens, *Aedes caspius Pallas*,* Ae. detritus* Haliday, *Ae. dorsalis* (Meigen), *Coquillettidia richiardii* Ficalbi, *Culiseta annulata* Schrank and *Cs. morsitans* (Theobald), *Aedes cinereus* Meigen, *Ae. cantans* Meigen and *Ae. punctor* Kirby [60]. Such data would not only improve understanding of ecological dynamics but also enhance the accuracy of disease risk assessments and inform more effective public health planning.

Overall, these results provide a robust foundation for future research and offer valuable evidence and ecological insight to inform vector surveillance and management strategies for diseases transmitted by mosquitoes.

## Supplementary Information

Supplementary Material 1.

## Data Availability

The datasets generated and analysed during the current study are publicly available in the VectorByte VecDyn repository at https://vectorbyte.crc.nd.edu/vecdyn-detail/27003.

## Abbreviations

Cx.: Culex
nRMSE: Normalised residual squared error
RCP: Representative Concentration Pathway
WNV: West Nile virus
UK: United Kingdom
