# Supplementary material for: Climate-based forecasting from national Culex mosquito surveillance to support West Nile and Usutu virus preparedness in England and Wales
Source: BMC Public Health. 2026 Jun 23;26:2531. doi: 10.1186/s12889-026-28033-5 (PMC13543362; doi:10.1186/s12889-026-28033-5)
Supplement: Supplementary file 1 [file 12889_2026_28033_MOESM1_ESM.docx]

# Supplementary Material

## Environmental covariates

ERA5-Land hourly data for 2023 and 2024 were downloaded from Copernicus Climate Data Store [1] for temperature, precipitation, wind, potential evaporation and soil moisture, which were processed to calculate annual, cold, dry, warm and wet quarter potential evaporation and soil moisture, 19 bioclimatic variables, annual number of dry days, annual daily maximum precipitation rate, June and July 2023 and 2024 mean daily precipitation rate, June and July 2023 and 2024 minimum, maximum and mean daily temperature, and June and July mean wind speed.

The corresponding future environmental covariates were also downloaded from Copernicus Climate Data Store from the “Downscaled bioclimatic indicators for selected regions from 1950 to 2100 derived from climate projections” dataset [2]. This provides environmental data derived from CMIP5 climate projections bias-adjusted against ERA5 reanalysis data. Projections were made using an ensemble of the HadGEM2-ES, bcc-csm1-1-m, GFDL-ESM2M, IPSL-CM5A-LR and NorESM1-M models for two future climate scenarios, Representative Concentration Pathway (RCP) 4.5 and RCP 8.5.

Additionally, elevation was downloaded from WorldClim 2.1, derived from SRTM elevation data [3], and the 2023 land cover map was downloaded from UKCEH [4], which was further processed to represent three categories: urban, suburban and rural (all remaining land cover classes which were not urban or suburban). Spatial resolutions for all covariates are described below.

The following table displays the initial environmental covariates considered for the models prior to analysis of collinearity and correlation between present and future data. Present day bioclimatic variables were manually calculated from the Copernicus ERA-5-Land hourly data. Some Copernicus ERA-5 Land hourly rasters did not extend over locations of surveillance points (near the coast), and therefore for these points, values were extracted from the nearest available pixel.

SM Table 1 Initial covariates considered for the models prior to correlation analysis. All Copernicus ERA-5-Land hourly data had a horizontal resolution of 0.1° x 0.1°, CMIP5 projections had a resolution of 1 km x 1 km, WorldClim elevation data had a resolution of 30 seconds, building count had a resolution of 500 m x 500 m, and land cover had a resolution of 10 m x 10 m. All were projected into the coordinate reference system WGS 84 and kept at their original resolution.

| **Abbreviation** | **Description** | **Present day source** | **Future projection source** |
| --- | --- | --- | --- |
| BIO01 | Annual Mean Temperature | Copernicus ERA-5-Land hourly | Copernicus CMIP5 ERA5-bias adjusted |
| BIO02 | Mean Diurnal Range (Mean of monthly (max temp - min temp)) | Copernicus ERA-5-Land hourly | Copernicus CMIP5 ERA5-bias adjusted |
| BIO03 | Isothermality (BIO2/BIO7) (×100) | Copernicus ERA-5-Land hourly | Copernicus CMIP5 ERA5-bias adjusted |
| BIO04 | Temperature Seasonality (standard deviation ×100) | Copernicus ERA-5-Land hourly | Copernicus CMIP5 ERA5-bias adjusted |
| BIO05 | Max Temperature of Warmest Month | Copernicus ERA-5-Land hourly | Copernicus CMIP5 ERA5-bias adjusted |
| BIO06 | Min Temperature of Coldest Month | Copernicus ERA-5-Land hourly | Copernicus CMIP5 ERA5-bias adjusted |
| BIO07 | Temperature Annual Range (BIO5-BIO6) | Copernicus ERA-5-Land hourly | Copernicus CMIP5 ERA5-bias adjusted |
| BIO08 | Mean Temperature of Wettest Quarter | Copernicus ERA-5-Land hourly | Copernicus CMIP5 ERA5-bias adjusted |
| BIO09 | Mean Temperature of Driest Quarter | Copernicus ERA-5-Land hourly | Copernicus CMIP5 ERA5-bias adjusted |
| BIO10 | Mean Temperature of Warmest Quarter | Copernicus ERA-5-Land hourly | Copernicus CMIP5 ERA5-bias adjusted |
| BIO11 | Mean Temperature of Coldest Quarter | Copernicus ERA-5-Land hourly | Copernicus CMIP5 ERA5-bias adjusted |
| BIO12 | Annual Precipitation | Copernicus ERA-5-Land hourly | Copernicus CMIP5 ERA5-bias adjusted |
| BIO13 | Precipitation of Wettest Month | Copernicus ERA-5-Land hourly | Copernicus CMIP5 ERA5-bias adjusted |
| BIO14 | Precipitation of Driest Month | Copernicus ERA-5-Land hourly | Copernicus CMIP5 ERA5-bias adjusted |
| BIO15 | Precipitation Seasonality (Coefficient of Variation) | Copernicus ERA-5-Land hourly | Copernicus CMIP5 ERA5-bias adjusted |
| BIO16 | Precipitation of Wettest Quarter | Copernicus ERA-5-Land hourly | Copernicus CMIP5 ERA5-bias adjusted |
| BIO17 | Precipitation of Driest Quarter | Copernicus ERA-5-Land hourly | Copernicus CMIP5 ERA5-bias adjusted |
| BIO18 | Precipitation of Warmest Quarter | Copernicus ERA-5-Land hourly | Copernicus CMIP5 ERA5-bias adjusted |
| BIO19 | Precipitation of Coldest Quarter | Copernicus ERA-5-Land hourly | Copernicus CMIP5 ERA5-bias adjusted |
| SOIL_ANN | Annual mean volumetric soil moisture | Copernicus ERA-5-Land hourly | Copernicus CMIP5 ERA5-bias adjusted |
| SOIL_COLD | Mean volumetric soil moisture of Coldest Quarter | Copernicus ERA-5-Land hourly | Copernicus CMIP5 ERA5-bias adjusted |
| SOIL_DRY | Mean volumetric soil moisture of Driest Quarter | Copernicus ERA-5-Land hourly | Copernicus CMIP5 ERA5-bias adjusted |
| SOIL_WARM | Mean volumetric soil moisture of Warmest Quarter | Copernicus ERA-5-Land hourly | Copernicus CMIP5 ERA5-bias adjusted |
| SOIL_WET | Mean volumetric soil moisture of Wettest Quarter | Copernicus ERA-5-Land hourly | Copernicus CMIP5 ERA5-bias adjusted |
| PEV_ANN | Annual mean potential evaporation | Copernicus ERA-5-Land hourly | Copernicus CMIP5 ERA5-bias adjusted |
| PEV_COLD | Mean potential evaporation of Coldest Quarter | Copernicus ERA-5-Land hourly | Copernicus CMIP5 ERA5-bias adjusted |
| PEV_DRY | Mean potential evaporation of Driest Quarter | Copernicus ERA-5-Land hourly | Copernicus CMIP5 ERA5-bias adjusted |
| PEV_WARM | Mean potential evaporation of Warmest Quarter | Copernicus ERA-5-Land hourly | Copernicus CMIP5 ERA5-bias adjusted |
| PEV_WET | Mean potential evaporation of Wettest Quarter | Copernicus ERA-5-Land hourly | Copernicus CMIP5 ERA5-bias adjusted |
| PREC_ANN | Annual maximum daily precipitation rate | Copernicus ERA-5-Land hourly | Copernicus CMIP5 ERA5-bias adjusted |
| PREC_1m | Mean monthly precipitation rate 1 month prior to surveillance (June) | Copernicus ERA-5-Land hourly | Copernicus CMIP5 ERA5-bias adjusted |
| PREC | Mean monthly precipitation rate in month of surveillance (July) | Copernicus ERA-5-Land hourly | Copernicus CMIP5 ERA5-bias adjusted |
| MAX_TEMP_1m | Maximum monthly temperature 1 month prior to surveillance (June) | Copernicus ERA-5-Land hourly | Copernicus CMIP5 ERA5-bias adjusted |
| MAX_TEMP | Maximum monthly temperature in month of surveillance (July) | Copernicus ERA-5-Land hourly | Copernicus CMIP5 ERA5-bias adjusted |
| AV_TEMP_1m | Mean monthly temperature 1 month prior to surveillance (June) | Copernicus ERA-5-Land hourly | Copernicus CMIP5 ERA5-bias adjusted |
| AV_TEMP | Mean monthly temperature in month of surveillance (July) | Copernicus ERA-5-Land hourly | Copernicus CMIP5 ERA5-bias adjusted |
| MIN_TEMP_1m | Minimum monthly temperature 1 month prior to surveillance (June) | Copernicus ERA-5-Land hourly | Copernicus CMIP5 ERA5-bias adjusted |
| MIN_TEMP | Minimum monthly temperature in month of surveillance (July) | Copernicus ERA-5-Land hourly | Copernicus CMIP5 ERA5-bias adjusted |
| WND_1m | Mean monthly wind speed in month prior to surveillance (June) | Copernicus ERA-5-Land hourly | Copernicus CMIP5 ERA5-bias adjusted |
| WND | Mean monthly wind speed in month of surveillance (July) | Copernicus ERA-5-Land hourly | Copernicus CMIP5 ERA5-bias adjusted |
| DEM | Elevation | WorldClim 2.1, derived from SRTM elevation data | WorldClim 2.1, derived from SRTM elevation data |
| BUILD_500m | Number of buildings within 500m radius | OpenStreetMap [5] | OpenStreetMap |
| RU | Landcover (rural, suburban, urban classification) | UKCEH Land cover 2023 | UKCEH Land cover 2023 |
| SPG_70 | Spatial cluster at 70km | Computed | Computed |

## Covariate pre-processing

To reduce collinearity in the covariates, covariate pairs were tested using a correlation matrix based on Pearson’s coefficients, where a collinearity was identified if $r$ > |0.7|. To determine which of the covariates is retained in the model, negative binomial generalised linear models (GLMs) were then fitted with response variables of either *Cx. p. pipiens*, *Cx. p. molestus* or *Cx. torrentium* abundance and each environmental covariate as the sole predictor in turn. The percentage of “deviance explained” ($DE$) was then calculated using the following formula,

$$DE=100*\frac{D_{null}-D_{res}}{D_{null}}$$

where $D_{null}$ is the null deviance and $D_{res}$ is the residual deviance. This was then scaled between 0 and 1 using the min-max scaling method. While univariate deviance explained does not fully capture multivariable relationships, it was used as a pragmatic screening tool to reduce dimensionality prior to multivariable modelling, rather than as a criterion for final inference. Additionally, an ecological weight score was assigned, where covariates had a neutral score of 3/5, apart from July monthly covariates of precipitation and mean, minimum and maximum temperatures, which were assigned a score of 5/5, because temporally close weather conditions to the trapping date summarised over 30 days, and previously identified as preferred covariates in *Culex* species modelling [6]. The percentage of “deviance explained” and the ecological scores were then combined with a weight of 70% “deviance explained” and 30% ecological score, to calculate the final importance score for each of the three response variables (*Cx. p. pipiens*, *Cx. p. molestus* or *Cx. torrentium* abundance).

Pairs of collinear covariates were then ranked in order of most correlated to least, and sequentially the covariate with the lowest importance score was removed each pair until no highly correlated pairs remained, and a list of potential environmental covariate candidates remained to be used in the model. This resulted in 11 covariates remaining for *Cx. torrentium* and 13 for *Cx. p. pipiens* and *Cx. p. molestus*.

## Final covariates after pre-processing

The following combinations of covariates were used in the variable selection via cross-validation approach to determine which abundance models performed the best:

| **Cx. p. pipiens** | **Cx. p. molestus** | **Cx. torrentium** |
| --- | --- | --- |
| SOIL_WARM | SOIL_DRY | SOIL_ANN |
| BIO02 | BIO02 | BIO07 |
| BIO04 | BIO09 | BIO09 |
| BIO09 | BIO11 | BIO14 |
| BIO14 | BIO14 | BIO15 |
| BIO15 | BIO15 | BIO18 |
| BIO18 | BIO17 | PREC_1m |
| PREC_1m | PREC | MAX_TEMP |
| MAX_TEMP | MAX_TEMP_1m | WND_1m |
| AV_TEMP_1m | MAX_TEMP | DEM |
| WND_1m | WND | BUILD_500m |
| DEM | DEM |  |
| BUILD_500m | BUILD_500m |  |

SM Figure 1 below displays the maps of the candidate variables for model selection.

SM Figure 1 Rasters of all ERA-5 covariates (mean of 2023 and 2024 data) used in the modelling of present day abundances

## References

1. Copernicus Climate Change Service (C3S). ERA5-Land hourly data from 1950 to present [dataset]. Copernicus Climate Change Service Climate Data Store (CDS); 2019. doi:10.24381/cds.e2161bac. Accessed December 1, 2024
2. Copernicus Climate Change Service (C3S). Downscaled bioclimatic indicators for selected regions from 1950 to 2100 derived from climate projections [dataset]. Copernicus Climate Change Service Climate Data Store (CDS); 2021. doi:10.24381/cds.0ab27596. Accessed December 1, 2024.
3. Fick SE, Hijmans RJ. WorldClim 2: new 1 km spatial resolution climate surfaces for global land areas. Int J Climatol. 2017;37(12):4302–4315.
4. Morton RD, Marston CG, O’Neil AW, Rowland CS. Land Cover Map 2023 (10 m classified pixels, GB) [dataset]. NERC EDS Environmental Information Data Centre; 2024. doi:10.5285/7727ce7d-531e-4d77-b756-5cc59ff016bd. Accessed December 1, 2024.
5. Humanitarian OpenStreetMap Team (HOT). GBR buildings polygons [dataset]. 2024. Available from: <https://data.humdata.org/dataset/hotosm_gbr_buildings>. Accessed September 1, 2025.
6. Ludwig A, Rousseu F, Kotchi SO, Allostry J, Fournier RA. Mapping the abundance of endemic mosquito-borne diseases vectors in southern Quebec. BMC Public Health. 2023 May 22;23(1):924.

## Model Selection Robustness via Cross-Validation

To evaluate robustness the robustness of abundance model selection, we repeated the entire model filtering procedure on 10 random subsamples of the data, each removing 10% of observations. Ensembles were then generated from the final chosen models and pixel values compared using Pearson’s correlation.

Ensemble prediction sets were highly correlated with the original ensemble prediction set for *Culex p. pipiens* (mean r = 0.88, range r = 0.85 to 0.91)*,* *Culex p. molestus* (mean r = 0.98, range r = 0.96 to 0.99)*,* and *Culex torrentium* (mean r = 0.93 , range r = 0.87 to 0.94), indicating that selected models are robust to sampling variation.

To further assess the robustness of our approach, we repeated the analysis without the conditions that selected models must have at least one significant covariate. In the *Culex p. pipiens* ensemble, all 100 formulae remained identical; in the *Culex p. molestus* ensemble, 78 formulae were consistent; and in the *Culex torrentium* ensemble, only 12 models overlapped – although this was the species with the least available data. Comparisons between ensembles showed very strong agreement: for *Culex p. molestus*, the ensembles were highly correlated (r = 0.99) with a residual mean square error of 0.01 mosquito, and for *Culex torrentium*, correlation was also high (r = 0.9) with a residual mean square error of 0.05 mosquito. These results demonstrate that, even with fewer overlapping models, the ensembles produced highly similar outcomes, underscoring the stability and robustness of the workflow.

While these analyses demonstrate that predictions are stable to model sampling variation and model filtering choices, such stability does not imply unbiased parameter estimation. Rather, it indicates that the predictive surface is robust under the assumptions and constraints of the modelling framework.

## Presence and absence model development

To take into account the low detection rate and limited temporal sampling effort, presence and absence models were developed for the three *Culex* species in England and Wales to explore the drivers of habitats suitability and likely areas where these mosquitoes may be found. The methodology for these models followed a similar outline to that in the main manuscript, however, prior to building the model, duplicate sites where surveillance recorded no mosquitoes in one year, and one or more mosquitoes in another year, were reduced to only keep the year positive for mosquitoes. Count data were then transformed into presence and absence data, represented as 0 if absent and 1 if present.

Models for confirmed *Cx. p. pipiens*, *Cx. p. molestus* or *Cx. torrentium* collected during this study at location $i$ were fitted by using generalized linear mixed model (GLMM) with a binomial distribution and a logit link function, implemented in the *glmmTMB* package.

The mean of the AUC values across the three iterations were then calculated to determine which 100 models of each mosquito performed the best and were to be used for further analysis, as per the abundance models.

## Presence and absence model results

When evaluated using a five-fold cross validation approach, including the model formulae with the spatial ID group random effect now added where required, the *Culex p. pipiens* presence models had a mean AUC 0.72 ± 0.06, the *Cx. p. molestus* models had a mean AUC of 0.84 ± 0.10, and the *Cx. torrentium* models had a mean AUC of 0.65 ± 0.09.

From the models included in the ensemble for each species, the percentage of significant covariates is presented in SM Table 2.

SM Table 2 Percentage of models with each significant covariate identified and their association with presence. Abbreviations can be found in Supplementary Material 1. A weak (+/-) association is where 50-70% of the models experienced a positive/negative association, a moderate (++/--) association is 70-90% and a strong (+++/---) association is >90%.

| Model | Covariates | Percentage of models significant | Association with presence |
| --- | --- | --- | --- |
| *Culex p. pipiens* | Maximum temperature (MAX_TEMP)  Wind speed (WND)  Elevation (DEM)  Soil moisture warmest quarter (SOIL_WARM)  Mean diurnal range (BIO02)  Precipitation coldest month (BIO14)  Temperature seasonality (BIO04)  Precipitation rate (PREC)  Mean temperature driest quarter (BIO09) | 99%  57%  28%  23%  15%  11%  5%  4%  1% | +++  ---  ---  ---  ---  +++  ---  +++  +++ |
| *Culex p. molestus* | Precipitation rate one month prior (PREC_1m)  Precipitation seasonality (BIO15) | 89%  44% | ---  --- |
| *Culex torrentium* | Wind speed one month prior (WND_1m)  Temperature annual range (BIO07) | 87%  1% | ---  +++ |

A previous modelling study in the UK identified that *Culex* (not specified which species) presence was most impacted by altitude [1] which was not what we observed. *Culex p. pipiens* showed high probability of presence across most of England, especially in the southeast regions. Areas with higher elevation, such as Wales and North Pennines showed a lower probability of presence. *Culex p. molestus and Cx. torrentium* displayed a much lower probability of presence in comparison to *Cx. p. pipiens*. The model predicted that *Culex p. molestus* would have a higher probability of presence in the southeast of England, especially around Greater London, Kent and Norfolk. Whereas, the *Cx. torrentium* model predicted increased probability of presence in Wales and the south of England (SM Figure 2).

SM Figure 2 Probability of presence of Cx. p. pipiens (TOP), Cx. p. molestus (MIDDLE), Cx. torrentium (BOTTOM) and their associated uncertainties.

## References

1. Simons RR, Croft S, Rees E, Tearne O, Arnold ME, Johnson N. Using species distribution models to predict potential hot-spots for Rift Valley fever establishment in the United Kingdom. PLoS One. 2019;14(12):e0225250.

## Maps with observations

The following maps (SM Figures 3-8) display the predicted abundance or probability of presence of the ensemble with field data overlaid.

SM Figure 3 Predicted present day Culex p. pipiens abundance with observed abundances from field data

SM Figure 4 Predicted present day Culex p. molestus abundance with observed abundances from field data

SM Figure 5 Predicted present day Culex torrentium abundance with observed abundances from field data

### Spatial boundaries

As observed in the *Culex torrentium* maps*,* a sharp spatial boundary artefacts appear as a result of the spatial group intercept in the model. A spatial group random intercept is a coarse approximation of a spatial process and can introduce artefacts at cluster boundaries. However, it provided a computationally efficient way to account for spatial dependence and reduce type I error, which was the primary concern. This approach ensured that residuals were appropriately corrected while remaining feasible to implement. Alternative methods were explored with more sophisticated spatial correlation structures, but convergence was consistently problematic. Given these constraints, the cluster-ID random intercept offered a practical compromise between statistical rigor and computational feasibility. To further validate this choice, we checked Moran’s I on the residuals after applying the cluster intercept to any model requiring it. These checks confirmed that spatial autocorrelation was substantially reduced, demonstrating that the approach achieved its intended purpose.

SM Figure 6 Predicted present day Culex p. pipiens probability of presence with observed catches from field data. X = present, O = absent

SM Figure 7 Predicted present day Culex p. molestus probability of presence with observed catches from field data. X = present, O = absent

SM Figure 8 Predicted present day Culex torrentium probability of presence with observed catches from field data. X = present, O = absent

## Maps with masked areas

The following maps display areas in grey which have been capped at the value of the 99^th^ percentile of the raw abundance in the main publication to prevent spurious results.

SM Figure 9 Predicted present day Culex p. pipiens abundance with masked out areas where results were above the 99^th^ percentile. These included along the river Thames, river Exe and the Severn estuary.

SM Figure 10 Predicted present day Culex p. molestus abundance with masked out areas where results were above the 99th percentile. These included along the river Thames, and small areas in Kent and Cornwall.

SM Figure 11 Predicted present day Culex torrentium abundance with masked out areas where results were above the 99th percentile. These included in the Cambridgeshire and Hertfordshire areas.

## Hybrid mosquito locations

Analysis of hybrid abundance and presence was not undertaken in this study to avoid unnecessary complexity in the models and challenges with inference. In addition, only 1^st^ generation hybrids can be identified, and therefore back-crosses will be identified as *Culex p. pipiens* or *Culex p. molestus* contributing further to the challenges associated with inference. The hybrid dataset was relatively small across the two years (15 points in 2023, contributing 22 mosquitoes, and 9 points in 2024, also contributing 22 mosquitoes, with 4 of the 2024 points being repeat locations from 2023).

By definition, hybrids are biological crosses between *Cx. p. pipiens* and *Cx. p. molestus* and are therefore expected in regions where both species co-occur. In both 2023 and 2024, *Cx. p. pipiens* was present in every trap that contained hybrids, whereas only 5 traps with hybrids also contained *Cx. p. molestus*, underscoring the challenges of capturing this species.

Supplementary Figure 12 presents the predicted distribution of *Cx. p. molestus* with hybrid catch locations overlaid. This figure illustrates the likely presence of *Cx. p. molestus* across southern England and Wales, extending beyond the southeast as originally predicted.

*SM Figure 12 Predicted present day Culex p. molestus probability of presence with observed catch locations of hybrids from field data. X = presence of hybrids, O = presence of molestus.*

Little is available in the literature about the hybridisation zone in Europe, however hybrids have been recorded in Germany [1], the Netherlands [2] and Portugal [3]. In Portugal, it was observed that hybrids occurred more frequently in the southern regions where *Culex p. molestus* occurred sympatrically with *Culex p. pipiens* above ground, whereas in the north, *Culex p. molestus* preferred subterranean regions [3].

The Portugese study was undertaken almost two decades ago, and since then further climate and environmental changes have occurred, resulting in a northern expansion of suitable vector habitats. It is plausible that this is now what we are observing in England, as our study only identified *Culex p. molestus* and hybrids (apart from one) in the south. The capture methods we used were not adaptable for subterranean habitats, and therefore further research would give a greater understanding into whether *Culex p. molestus* was truly absent in the north, or subterranean.

## References

1. Rudolf M, Czajka C, Börstler J, Melaun C, Jöst H, von Thien H, Badusche M, Becker N, Schmidt-Chanasit J, Krüger A, Tannich E. First nationwide surveillance of Culex pipiens complex and Culex torrentium mosquitoes demonstrated the presence of Culex pipiens biotype pipiens/molestus hybrids in Germany. PloS one. 2013 Sep 11;8(9):e71832.

2. Reusken CB, De Vries A, Buijs J, Braks MA, Den Hartog W, Scholte EJ. First evidence for presence of Culex pipiens biotype molestus in the Netherlands, and of hybrid biotype pipiens and molestus in northern Europe. Journal of Vector Ecology. 2010 Jun;35(1):210-2.

3. Gomes B, Sousa CA, Novo MT, Freitas FB, Alves R, Côrte-Real AR, Salgueiro P, Donnelly MJ, Almeida AP, Pinto J. Asymmetric introgression between sympatric molestus and pipiens forms of Culex pipiens (Diptera: Culicidae) in the Comporta region, Portugal. BMC Evolutionary Biology. 2009 Nov 6;9(1):262.

## Scatter diagrams of maps and abundance observations

Scatter plots were generated by comparing the final ensemble maps with observed locations, log‑transformed as described in the main text to aid interpretation on a more manageable scale.

The plots indicated that the maps captured the overall trend in *Culex p. pipiens* abundance, though variation was evident across the full range of values. Predictions for *Culex p. molestus* and *Culex torrentium* were less consistent, with positive abundance values often lower than those observed. However, because the absolute ranges of values for these species are small, the apparent discrepancies can give an inflated impression of uncertainty relative to their true magnitude.

*SM Figure 13 Scatter plots of log10(x+1) transformed mosquito abundance predicted and observed mosquito abundance values. The red dotted line represents the perfect prediction.*

## Geographical locations

*SM Figure 14 Map of Culex pipiens pipiens abundance and locations of the estuaries highlighted in the main manuscript*

*SM Figure 15 Map of geographical regions described in the manuscript text. Counties and Unitary Authorities (December 2024) Boundaries UK BFC shapefile available from https://geoportal.statistics.gov.uk/search?q=BDY_CTY%202024&sort=Title%7Ctitle%7Casc*
